# Presence of anti-IgLON5 antibody in a case of sporadic Creutzfeldt–Jakob disease with sleep disturbance as a prominent symptom

**DOI:** 10.1007/s10072-022-06434-9

**Published:** 2022-10-06

**Authors:** Zhongyun Chen, Jing Zhang, Yu Kong, Haitian Nan, Li Liu, Lin Wang, Shi Qi, Liyong Wu

**Affiliations:** 1 Department of Neurology, Xuanwu Hospital, Capital Medical University, Beijing, 100053 China; 2 State Key Laboratory for Infectious Disease Prevention and Control, National Institute for Viral Disease Control and Prevention, Chinese Center for Disease Control and Prevention, Beijing, 102206 China

Dear Editor ,

Creutzfeldt–Jakob disease (CJD) is a rare, fatal, degenerative prion disease commonly presenting with rapidly progressive dementia. The differential diagnosis of it includes but is not limited to, autoimmune, infectious, neoplastic, or neurodegenerative diseases. Among these, distinguishing between CJD and autoimmune encephalopathy can be challenging, especially with the co-occurrence of clinicoradiological or laboratory features for both diseases. Less than 5% of CJD patients could produce autoantibodies, including those specific for NMDAR, GluRε2, VGKC complex, CASPR2, and GLyR; those antibodies may also be a factor in the development of the disease’s clinical symptoms. The anti-IgLON5 antibody, first described and characterized in 2014, targets a surface or synaptic protein of uncertain function. Anti-IgLON5 disease, individuals carrying this anti-IgLON5 antibody, exhibits sleep disorders, gait abnormalities, bulbar dysfunction, and cognitive dysfunction [1]. The coexistence of anti-IgLON5 antibody in CJD has not been reported. Herein we report the first case of sporadic CJC (sCJD) with sleep disturbance as a prominent symptom and a positive anti-IgLON5 antibody.

## Case presentation

A 69-year-old woman presented with worsening dementia, sleeplessness, and abnormal sleep behaviors in September 2020, and drugs that were prescribed to boost cognition and sleep were ineffectual. She began to exhibit behavioral problems such as inhibition, apathy, and stereotyped speech, as well as ataxia, uneven walking, motor delays, walking with help, and incontinence 8.5 months after the initial symptoms. Her family medical history was unremarkable. The patient was admitted to our hospital 10 months after the onset of symptoms. Neurological examination revealed severe cognitive impairment (1/30 on the Mini-Mental State/Examination), increased muscle tension in the extremities, normal deep tendon reflex, and negative pathological reflexes. Other neurological tests were not performed.

**Fig. 1:**
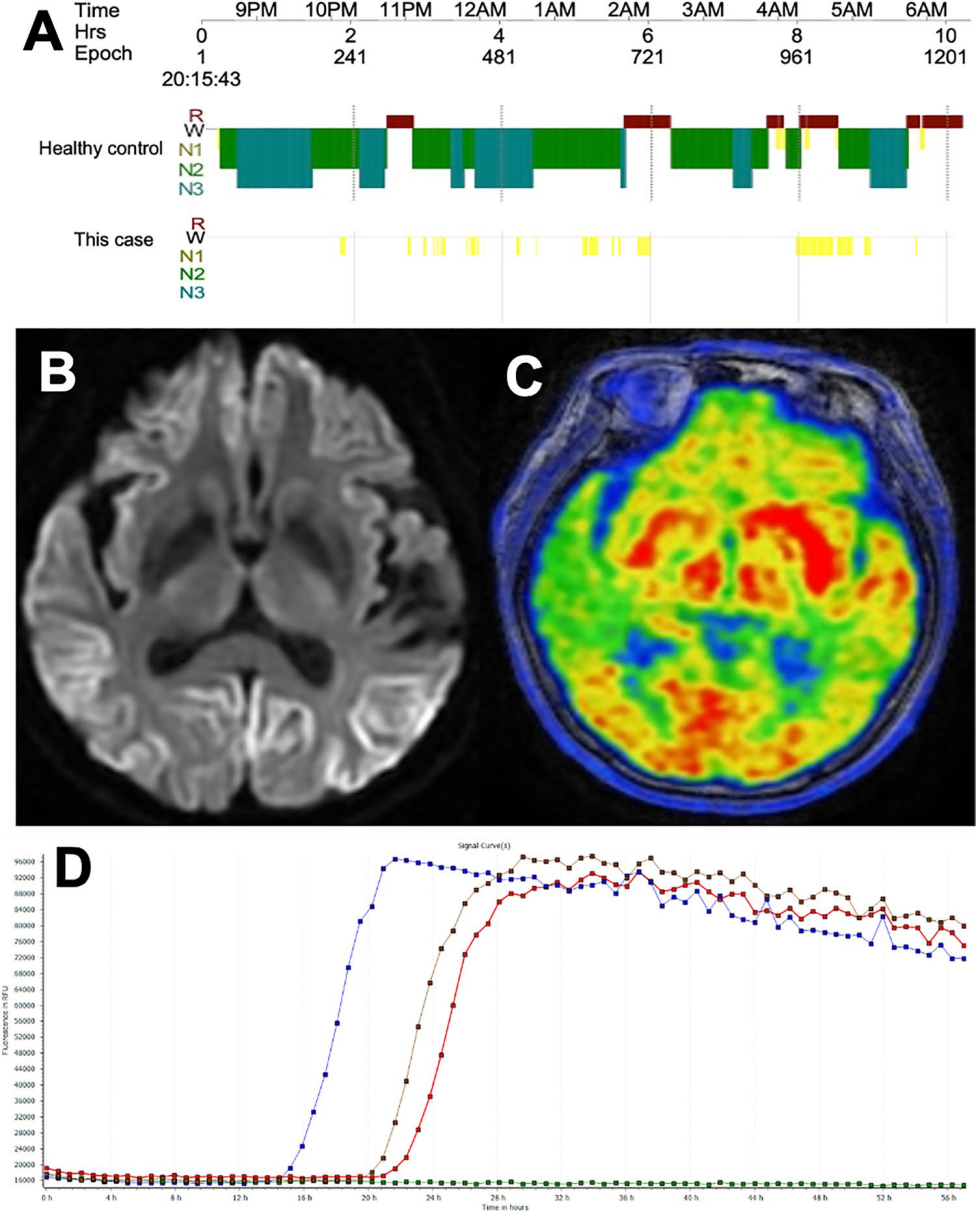
**A** The polysomnogram showed a decrease in total sleep time and an increase in sleep segments, with the patient being in stage N1 all night and not seeing stages N2 or N3 and R. **B** Cranial magnetic resonance imaging (MRI) demonstrated bilateral frontotemporal, parietal, and occipital lobe hyperintensity. **C** The ^18^F-fluorodeoxyglucose positron emission tomography (FDG PET) showed significant hypometabolism in bilateral frontotemporal and parietal. **D** Four replicate reactions of patient CSF samples were used in the RT-QuIC. Each line represents the reaction curve of any sample in four repeats. A sample was considered to be positive when ≥ 2 wells revealed positive reaction curves

Paraneoplastic and neuronal antibodies were detected from blood and cerebrospinal fluid (CSF), and anti-IgLON5 antibodies were detected in the serum (titer 1:100 and 1:320 in a confirmation test) but not in the CSF. Polysomnography (PSG) revealed a reduction in total sleep time, a decrease in sleep efficacy (16.4%), and an increase in sleep fragmentation (Fig. 1A). Due to the drastically reduced duration of sleep, the possibility of sleep-disordered breathing could not be ascertained. The patient was a carrier of *HLA-DQB1*^*∗*^*05:01* but not the *HLA-DRB1*^*∗*^*10:01* allele. Cranial magnetic resonance imaging (MRI) demonstrated bilateral frontotemporal, parietal, and occipital lobe hyperintensity (Fig. 1B). The ^18^F-fluorodeoxyglucose positron emission tomography (FDG PET) also showed significant hypometabolism in these areas except for the occipital lobe (Fig. 1C). Electroencephalography (EEG) revealed periodic sharp wave complexes at a frequency of 2 Hz. The CSF A1-42/A1-40 ratio and phosphorylated tau concentrations were within normal limits (0.20 and 53.9 pg/mL, respectively), while the CSF total tau concentration (> 1321 pg/ml, normal range 290 pg/ml) dramatically increased. The CSF was negative for the 14–3-3 protein and positive for the real-time quaking-induced conversion (RT-QuIC) assay (Fig. 1D). Genetic sequencing did not reveal any pathogenic variants of the prion protein gene and the patient was homozygous for methionine/methionine at codon 129. The diagnosis of probable sCJD was made according to the WHO diagnostic criteria.

During hospitalization, the patient was treated with a high intravenous dosage of gamma globulin (0.4 g/kg/d for 5 days), methylprednisolone (500 mg/day for 5 consecutive days), and rituximab (600 mg). While the treatment reduced muscle tension to normal, the other symptoms were not significantly improved. Eleven months after the onset of the first symptoms, the patient exhibited bulbar dysfunction that manifested as dysphagia and inability to eat and was given nasal feeding. The patient had akinetic mutism and was incontinent and intranasally fed for the following 12 months. The patient’s family denied permission for further examination.

## Discussion

To the best of our knowledge, this is the first case report of probable sCJD with positive anti-IgLON5 antibody. Coincidentally, the patient also had a serious sleep disorder. It is well known that sleep disturbances are a prominent symptom of anti-IgLON5 disease, manifested as rapid eye movement sleep behavioral disorder, non-rapid eye movement sleep parasomnias, obstructive sleep apnea syndrome, and stridor [2]. However, these sleep-related symptoms have also been reported in 49–89% of CJD patients [3]. Rapidly progressive dementia is a prominent feature of CJD, while cognitive impairment has been reported in 53.8% of anti-IgLON5 disease cases [4]. It is therefore difficult to distinguish between the two disorders based on clinical symptoms alone.

Distinguishing between autoimmune encephalopathy and CJD can be a challenge. Geschwind et al. reported that 60% of cases in a cohort of 15 patients with VGKC complex antibody autoimmunity met the WHO diagnostic criteria for CJD [5]. Conversely, Peter et al. reported that approximately 6% (22/384) of patients with suspected CJD have autoimmune encephalitis at autopsy [6]. Given the lack of established disease-modifying treatment for CJD, immune therapy can differentiate between autoimmune encephalopathy and CJD. However, unlike other autoimmune encephalitis, only 36.5% of the patients with anti-IgLON5 disease that received immunosuppressants or immunomodulatory drugs achieved a sustained clinical response [7].

Less than 5% of CJD patients produce autoantibodies, but often at titers below 1:100 [8]. The low levels of these serum antibodies are presumably the result of extensive and rapid neuronal destruction. Although autoantibodies found in patients with sCJD are unlikely to be a major causative factor of neurodegenerative disease, they may contribute to the clinical symptoms during the course of the disease. The relatively low antibody titers in this patient raise the possibility of a false-positive result. However, given that this patient is a carrier of *HLA-DQB1*^*∗*^*05:01*, which is recognized in approximately 90% of patients with anti-IgLON5 antibodies and in only 3.38% of the Chinese population [9], and the prolonged disease course, the possibility of coexistence of the two diseases cannot be completely ruled out. There is evidence that the production of anti-IgLON5 antibodies could be a consequence of neurodegeneration [10]. We hypothesize that the scrapie isoform of the prion protein deposits in this patient triggered phosphorylated tau deposition, which coupled with a genetic susceptibility to anti-IgLON5 disease, eventually resulted in the positive of anti-IgLON5 antibody.

In conclusion, this case broadens the spectrum of neuronal antibodies in patients with CJD, although it is unclear whether this coexistence is a coincidence or a consequence. Screening for anti-IgLON5 antibody should be performed in future studies in patients with CJD, especially those with sleep disturbances.

## Data Availability

The datasets used during the current study are available from the corresponding author on reasonable request.

## Abbreviations

CJD: Creutzfeldt–Jakob disease
CSF: Cerebrospinal fluid
DWI: Diffusion-weighted image
EEG: Electroencephalogram
FLAIR: Fluid-attenuated inversion recovery
HLA: Human leukocyte antigen
MRI: Magnetic resonance imaging
PCR: Polymerase chain reaction
PRNP: Prion protein gene
PrP^Sc^: Scrapie isoform of the prion protein
PSWC: Periodic sharp wave complexes
RT-QuIC: Real-time quaking-induced conversion
RBD: Rapid eye movement sleep behavioral disorder
